# Ninein isoform contributions to intracellular processes and macrophage immune function

**DOI:** 10.1016/j.jbc.2025.108419

**Published:** 2025-03-18

**Authors:** Safia Omer, Elizabeth Persaud, Safia Mohammad, Bolu Ayo-Farinloye, Rebecca E. Heineman, Emily Wellwood, G. Adam Mott, Rene E. Harrison

**Affiliations:** Department of Biological Sciences, University of Toronto Scarborough, Toronto, Ontario, USA

**Keywords:** microtubule organization, centrosome cohesion, phagocytosis, dynein adaptors, isoforms and differential functions

## Abstract

Ninein is a multifunctional protein involved in microtubule (MT) organization and dynein/dynactin complex recruitment and activation. Several isoforms of ninein have been identified in various tissues, however, their relative contribution(s) are not clear. Here, we identify two ninein isoforms in mouse macrophages with distinct C-termini and disproportionate expression levels; a canonical ninein (ninein^CAN^) isoform and ninein isoform 2 (ninein^ISO2^). Analysis of ninein pre-mRNA exon-intron boundaries revealed that ninein^ISO2^ transcript is likely generated by two alternative splicing site selection events predicted to result in a distinct 3D structure compared to ninein^CAN^. We used selective and total protein knockdown experiments to assess the intracellular and functional roles of ninein in macrophages. Live cell imaging analyses of macrophages implicated both isoforms in regulating cell proliferation. MT regrowth following nocodazole depolymerization showed that both isoforms contributed to MT nucleation and structural integrity of the centrosome, as cells lacking ninein^CAN^ or ninein^ISO2^ contained multiple ectopic **γ**-tubulin foci. However, ninein^CAN^, but not ninein^ISO2^, was important for the separation of duplicated centrosomes during cell division. Despite a requirement of both ninein isoforms to recruit dynein/dynactin to the centrosome, only ninein^CAN^ was required for Golgi positioning and morphology, dynein-dependent events. We additionally found that ninein^ISO2^ was the primary isoform required for F-actin recruitment during the internalization of IgG-opsonized particles. Our study indicates that alternative splicing promotes both redundant and differential activities for ninein in MT organization, organelle positioning, and macrophage function.

The centrosome is a key cellular structure that consists of a pair of centrioles surrounded by dense pericentriolar material (PCM), which is essential for microtubule (MT) nucleation, anchorage, and radial organization. The MT cytoskeleton emanates from the centrosomes as a dense network of polarized filaments, with rapidly growing plus ends, and minus ends that are capped and anchored at the centrosome. The polarized MT arrays serve as tracks for motor complexes such as kinesin, which drives anterograde intracellular transport, and the minus end-directed motor dynein, which mediates retrograde intracellular transport and positioning of cargo including organelles (1, 2, 3, 4).

Ninein is a multifunctional protein that localizes to the subdistal appendages of the mother centriole and forms discrete speckles that move bidirectionally along MTs (5, 6, 7, 8). Ninein-knockout mice have a defective epidermal barrier (9), and bone deformation (10) and mutated forms of ninein have been associated with Seckel syndrome, which is characterized by dwarfism and cognitive deficiencies (11, 12). In studies of mouse forebrain and forebrain organoids, ninein was shown to play a major role in neuronal progenitor proliferation and differentiation (13, 14, 15, 16).

The cellular mechanism(s) leading to the developmental defects caused by ninein deficiency are not fully understood; however, several mechanisms are potentially involved. Ninein anchors MT minus ends at the centrosome (5, 8, 17, 18, 19, 20). Depletion of ninein or overexpression of dominant-negative ninein truncations leads to reduced MT nucleation (13, 20, 21, 22) and defective organization of the MT radial network (19). Disrupting ninein localization and activity is also linked to defective non-centrosomal MT arrays in various cell types, including polarized intestinal TC7 cells, polarized MDCKII cells, differentiating keratinocytes, and neurons (9, 23, 24). Depletion of ninein is also associated with premature separation of duplicated centrosomes (21) and impaired segregation and inheritance of the centrosome during cell division (14). These diverse activities are mediated by direct, and indirect, interactions of ninein with: PCM proteins (15, 25), F-actin regulators (23, 25), MT minus end and MT plus end proteins (23, 25), and dynein/dynactin complex subunits (25, 26, 27). How ninein interacts with all these proteins is not clear, but alternative splicing of ninein pre-mRNA likely increases its interactome. Ninein has a complex exon-intron organization and undergoes alternative splicing in mice (15, 28, 29) and humans (22, 30), resulting in cell-type and tissue-specific isoforms that lack central exons and/or contain variable C-termini. The best documented alternative splicing event occurs during neuron differentiation (15, 29), where the canonical centrosomal isoform of ninein is switched to a shorter isoform lacking central and C-terminus sequences, leading to a cytoplasmic ninein localization (15). However, the mechanism of ninein alternative splicing, and the role(s) of ninein isoforms in regulating cytoskeletal- and motor-dependent activities is unclear.

In this study, we quantified and characterized ninein isoforms in macrophages, a proliferative and morphological dynamic innate immune cell. We identified two ninein isoforms in mouse macrophages with different predicted structures. Using live cell imaging and immunostaining analysis, we found that both ninein^CAN^ (canonical ninein isoform, NCBI Accession: AAS87211) and ninein^ISO2^ (ninein isoform 2, NCBI Accession: NP_032723/Q61043-1) play roles in cell proliferation, with ninein^CAN^ potentially regulating the separation of duplicated centrosomes. We also found that both isoforms are centrosome-localized proteins that are essential for intact MT anchorage activity, maintaining a focused MT nucleation site, and dynein recruitment to the centrosome. Distinct activities were observed for ninein^CAN^ in Golgi positioning and morphology. Additionally, we showed that ninein^ISO2^ is the primary isoform required for the early stages of F-actin cortical recruitment following the binding of macrophages to IgG-coated targets. Together, these findings highlight the distinct and complementary roles of ninein isoforms in regulating MT nucleation and anchorage, centrosome integrity, and dynein-mediated activities.

## Results

### Identification of two ninein isoforms in mouse macrophages

**Figure 1:**
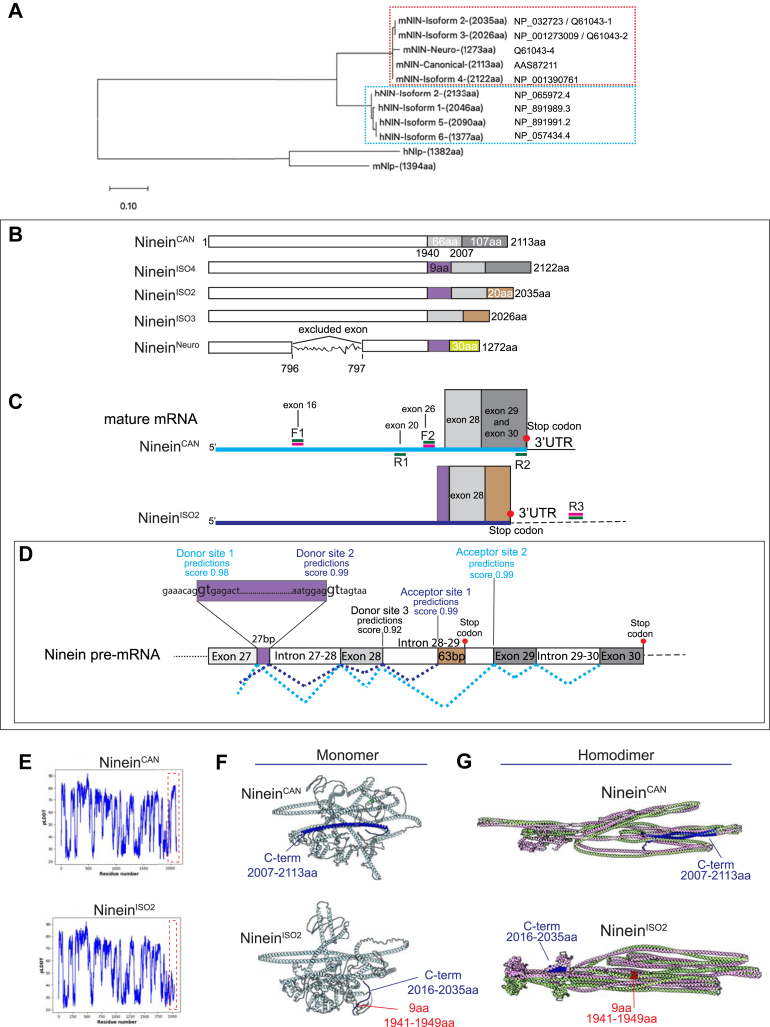
**Two isoforms of ninein are expressed in murine macrophages.***A*, unrooted phylogenetic tree of full-length protein sequences of ninein isoforms in mouse (mNIN), human (hNIN), and the paralogs mouse and human Ninein-Like protein (mNlp and hNlp, respectively). The phylogenetic tree was generated using maximum-likelihood analysis. Isoform amino acid (aa) length and accession numbers from NCBI and/or UniProt are shown. Branch lengths represent the sequence distance, with the scale bar of 0.1 corresponding to one aa substitution per 100 aa. *B*, schematic representation of protein-encoding ninein isoforms expressed in mouse, illustrating diverse C-terminus regions. Identical protein sequence segments are depicted in the same color and are not drawn to scale. *C*, diagram of ninein^CAN^ and ninein^ISO2^ mature mRNA. The diagram includes binding sites for forward (F) and reverse (R) primers used for cDNA amplification (*green bars*) and sequencing (*magenta bars*). Variable 3′UTRs are shown as *solid and dashed lines*. *D*, schematic of mouse ninein pre-mRNA encompassing sequences beyond exon 27. Predicted donor and acceptor splice sites are shown only for events involved in the formation of ninein^CAN^ and ninein^ISO2^. *Cyan* and *blue dotted lines* connect ninein^CAN^ and ninein^ISO2^ splicing junctions, respectively. Exons and introns are not drawn to scale. *E*, alphaFold2 analysis of ninein^CAN^ and ninein^ISO2^ full-length protein sequences. The plot shows the aa positions against the predicted local distance difference test (pLDDT) scores. *F*, predicted AlphaFold3 models of monomeric ninein^CAN^ and ninein^ISO2^. For ninein^CAN^, the C-terminus fragment (2007–2113 aa) is shown in *blue*. For ninein^ISO2^, the 9 aa motif (1941–1949 aa) is shown in *red*, and the variable C-terminus fragment (2016–2035 aa) is shown in *blue*. *G*, predicted three-dimensional structures of ninein^CAN^ and ninein^ISO2^ homodimers. Each monomer is colored differently. The variable C-terminal fragment of ninein^CAN^ is shown in *blue*. For ninein^ISO2^, the 9-amino acid motif is shown in *red*, and the variable C-terminus fragment is in *blue*.

Ninein is a critical MT-regulating protein, yet ninein isoform expression and how they relatively contribute to mouse macrophage function remains unclear. To examine ninein isoform diversity, we performed phylogenetic analysis of its alternatively spliced variants annotated in NCBI, Ensembl, and UniProt databases. A maximum-likelihood phylogenetic tree was constructed using multiple protein sequence alignments of five full-length ninein mouse isoforms (mNIN) and four isoforms of the human ninein (hNIN) to evaluate the cross-species conservation (Fig. 1*A*). This analysis revealed two clusters that represent an evolutionarily divergent ninein in mouse and human (Fig. 1*A*). Within human and mouse datasets, ninein isoforms have significant sequence homology, with increased diversity observed at the carboxyl (C)-terminus region (Fig. 1, *A* and *B*). The shortest ninein isoforms, isoform 6 in humans and ninein^Neuro^ (ninein neuron, UniProt: Q61043-4) in mice, each have a variable C-terminus, and lack a central exon encoding 713 and 707 amino acids (aa), respectively.

**Sup Figure 1:**
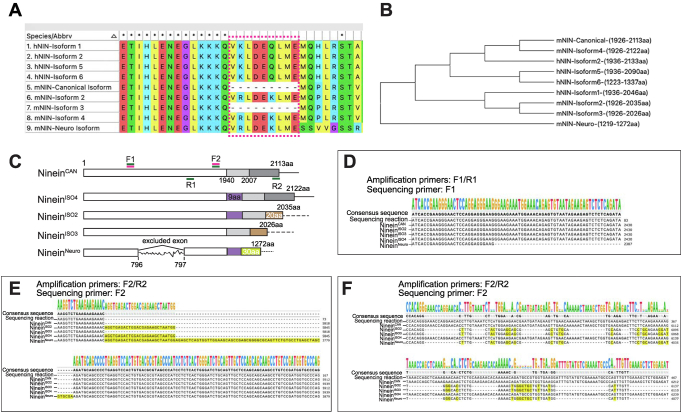


To elucidate the mechanism of alternative splicing that facilitates the formation of ninein isoforms in mice, we analyzed the relationship between splicing sites and the order of exons in the ninein protein sequence (Fig. 1, *C* and *D*). Splice site detection analysis (31) was carried out using the ninein genomic sequence following exon 27 (Fig. 1*D*). We found that the 3′end of exon 27 included two donor splice sites (prediction score 0.98 and 0.99, respectively, Fig. 1*D*), which contained the canonical GT/AG splice sites (32, 33). The usage of the first splice site would result in the formation of ninein^CAN^ and ninein^ISO3^ (ninein isoform 3, NCBI Accession: NP_001273009) transcripts, while the usage of the second donor site would lead to the inclusion of 27 bp translated into a unique motif of 9 aa VRLDEKLME present in ninein^ISO2^, ninein^ISO4^ (ninein isoform 4, NCBI Accession: NP_001390761), and ninein^Neuro^. Remarkably, sequence alignment of mNIN and hNIN variants showed that all human ninein isoforms contained a near identical 9 aa motif VKLDEQLME, flanked by highly conserved sequences in both species (Fig. S1*A*). Splice site detection analysis also revealed the presence of an acceptor site located in the middle of intron 28 to 29 (Fig. 1*D*). Usage of this site caused a premature termination codon, leading to the formation of the unique 20 aa at the C-terminus and an alternative 3′untranslated region (UTR) found in ninein^ISO2^ and ninein^ISO3^ isoforms (Fig. 1*D*). Intriguingly, phylogenetic analysis of the C- terminus sequences of mNIN and hNIN, revealed that mouse ninein^CAN^ and ninein^ISO4^ are closely clustered with the hNIN isoforms 2 (NCBI Accession: NP_065972.4), 5 (NCBI Accession: NP_891991.2) and 6 (NCBI Accession: NP_057434.4), while ninein^ISO2^ and ninein^ISO3^ are closely clustered with the hNIN isoform 1 (NCBI Accession: NP_891989.3) (Fig. S1*B*). Multiple sequence alignment of mouse ninein isoforms proteins revealed six N-terminus and central missense variants in exons 16 and 17 (K675E, E909K, Y1084S, Q1085R, K1155E, P1327A). These genetic variants are differentially present in mouse genetic backgrounds, as previously identified (28).

**Sup Figure 2:**
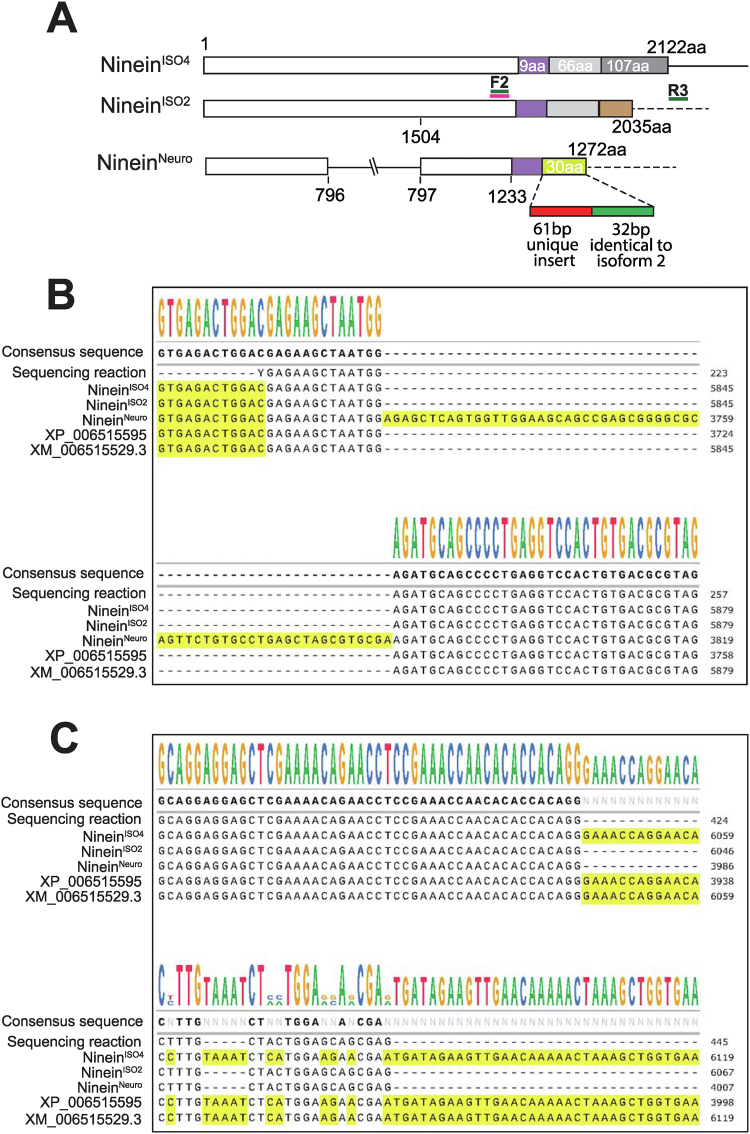


To analyze ninein transcripts in mouse macrophages, cDNA was synthesized from total RNA isolated from the murine macrophage-like cell line, RAW 264.7 (RAW) cells. Fragments of ninein cDNA were amplified, gel purified, and sequenced (primers are listed in Table 1). Amplification using F1/R1 primer pairs revealed a sequence read that corresponds to ninein^CAN^, ninein^ISO2^, ninein^ISO3^, and ninein^ISO4^ but not ninein^Neuro^ (Fig. S1, *C* and *D*). To narrow down ninein isoforms expressed in RAW cells, we used primer pairs targeted to C- terminus regions and 3′UTRs (Fig. 1*C*). F2/R2 amplification product confirmed the presence of a C- terminus sequence corresponding to ninein^CAN^ but not ninein^ISO2^, ninein^ISO4^, ninein^Neuro^ (Fig. S1*E*), or ninein^ISO3^ (Fig. S1*F*). Ninein^CAN^ was initially isolated from mouse peritoneal macrophages (5) and characterized in other cell types (6, 7, 20). Sequencing of the F2/R3 amplification product confirmed the presence of ninein^ISO2^ (Fig. S2, *A*–*C*), revealing a sequence read with a unique 27 bp sequence that is present in a subset of ninein isoforms (ninein^ISO2^, ninein^ISO4^, and ninein^Neuro^) along with a ninein^ISO2^-specific sequence at the C-terminus. Interestingly, protein sequence alignment of the C-terminus regions - specifically the last 95 aa of ninein^ISO2^ and the last 173 aa of ninein^CAN^ revealed a 39.96% identity and a 52.75% gap. In contrast, alignment of the 95 aa C-terminal end of ninein^ISO2^ and 96 aa C-terminus end of hNIN isoform 1 revealed a strong 86.46% identity with a 1.04% gap, suggesting cross-species conservation of the splicing event leading to the formation of the C-terminal polypeptide. While we cannot rule out the presence of additional ninein isoforms in mouse macrophages, our analysis confirmed the presence of two prevalent C-terminally variable isoforms: ninein^CAN^ and ninein^ISO2^.

**Table 1:** Primer sequence (5′-3′)

F1 5′AGGAGAAGCATGCTCTAGAG 3′	R1 5′TATTTTCTGAATCCAACTGTTGG 3′
F2 AGAGAGAGTGTGAACAGTCTC 3′	R2 5′ CTATGACCTCAGAGGAGGC 3′
	R3 5′TGGTAACAGTCAAATATTTT3′
qF1 5′AGCAGCTATTGGAAGAAAACG3′	qR1 5′ AAATTTCAGTAGCCATCT 3′
qF2 5′ AACTAAAGCTGGTGAAGAGAC 3′	qR2 5′ CTCCAGAGCTTTCAACAACT 3′
qF3 5′ACTGGACGAGAAGCTAATGG3′	qR3 5′AAGCTGCAGAAACTGCTC3′
F4 5′TTAGTGAACCGTCAGATCCGGCCACCATGGTGAGCAA3′	R4 5′GGTCTGCAATCTCACTCCGAAGCTCG3′
F5 5′TCGGAGTGAGATTGCAGACCTGGAAGG3′	R5 5′ GCAGCAGCTGCTTTCTTTCCGTGCTCAGGAT3′
F6 5′GGAAAGAAAGCAGCTGCTGCAAGACCTG3′	R6 5′TCTGCTGCAACCTGGAAATTTCAGTAGCCA3′
F7 5′AATTTCCAGGTTGCAGCAGAGGCTGAAG3′	R7 5′CGGTTTTGCACTCCTGCCTCTGAACAG3′
F8 5′ GAGGCAGGAGTGCAAAACCGAGGCGT 3′	R8 5′CCTGCAGGGCAGCCCTTCCCAGCAC3′
F9 5′GGGAAGGGCTGCCCTGCAGGGTG3′	R9 5′CTCCATTAGCTTCTCGTCCAGTCTCACCTGTTTCTTCTTCAGACCTTCG 3′
F10 5′GTGAGACTGGACGAGAAGCTAATGGAGATGCAGCCCCTGAGG3′	R10 5′GCAGAATTCGAAGCTTGAGCAGTACCTTCTTTTAAAAATAATTAACAACATATG3′

Next, we used AlphaFold2 to evaluate the secondary and tertiary structure of ninein^CAN^ and ninein^ISO2^. For both isoforms, the prediction of local conformation of the polypeptide chains of ninein^CAN^ and ninein^ISO2^ showed a dominance of coiled structural elements across the entire protein sequence, with high-confidence levels of pLDDT > 80 (Fig. 1*E*). However, the region spanning 1941 to 2035 aa of ninein^ISO2^, which corresponds to the sequence between the unique 9 aa motif and the stop codon, had no well-defined structure (Fig. 1, *E* and *F*), potentially forming a flexible or disordered conformation. Reconstituted 3D structural models of full-length monomers of ninein^CAN^ and ninein^ISO2^ revealed globular subunits composed predominately of α-helices coiled coils, with global structural differences between the two ninein isoforms (Fig. 1*F*). Like other dynein adaptors, ninein is predicted to form a homodimer (34, 35). AlphaFold3 modeling homodimer of ninein^CAN^ and ninein^ISO2^ isoforms predicted models featuring extended conformations with pronounced structural changes between the two protein variants (Fig. 1*G*). Collectively, the variable C-terminus sequences were sufficient to cause significant structural rearrangements that separate ninein^CAN^ and ninein^ISO2^.

### Ninein^CAN^ and ninein^ISO2^ isoforms accumulate at the centrosome in macrophages

**Figure 2:**
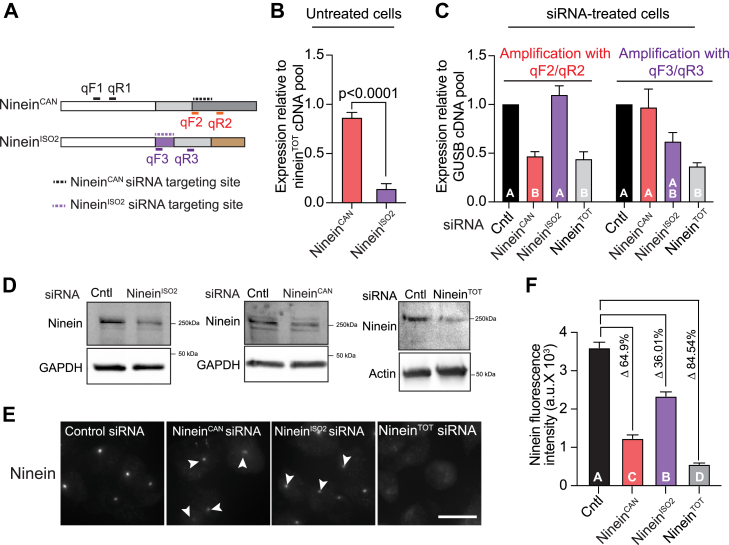
**Ninein^ISO2^ is expressed in RAW cells at lower levels compared to ninein^CAN^.***A*, diagram illustrating the binding sites for primers (shown as *solid bars*) used in RT-qPCR and isoform-siRNA target sites (shown as *dashed lines*). Primers were designed to amplify total ninein (qF1/R1) and to amplify specific ninein isoforms: ninein^CAN^ (qF2/R2) and ninein^ISO2^ (qF3/R3). *B*, quantification of ninein^CAN^ and ninein^ISO2^ expression relative to total ninein cDNA. RT-qPCR was conducted using four independent cDNA samples, with each qPCR reaction repeated in triplicate. *C*, quantification of ninein^CAN^ and ninein^ISO2^ expression relative to the internal housekeeping transcript GUSB. RT-qPCR was conducted using three different cDNA samples, and qPCR reactions were carried out in triplicate. *D*, representative immunoblots of total cell lysates from RAW cells treated with ninein^TOT^, ninein^CAN^ or ninein^ISO2^ siRNA, or control siRNA, probed for ninein and the loading controls GAPDH or β-Actin. *E*, confocal images of RAW cells transfected with ninein^TOT^, ninein^CAN^, ninein^ISO2^, or control siRNA, fixed and immunostained for ninein. Arrowheads mark ninein localization at the centrosome. *F*, quantification of ninein fluorescence intensity in RAW cells treated with the indicated siRNAs (n ≥ 78). *p*-values were calculated using a two-tailed Student's *t* test (*B*) and one-way ANOVA followed by Tukey's multiple comparisons test (from three independent experiments *C* and *F*). Letters denote significant differences between treatments (*p* < 0.05). Error bars represent the standard error of the mean (SEM). Scale bars = 10 μm.

**Sup Figure 3:**
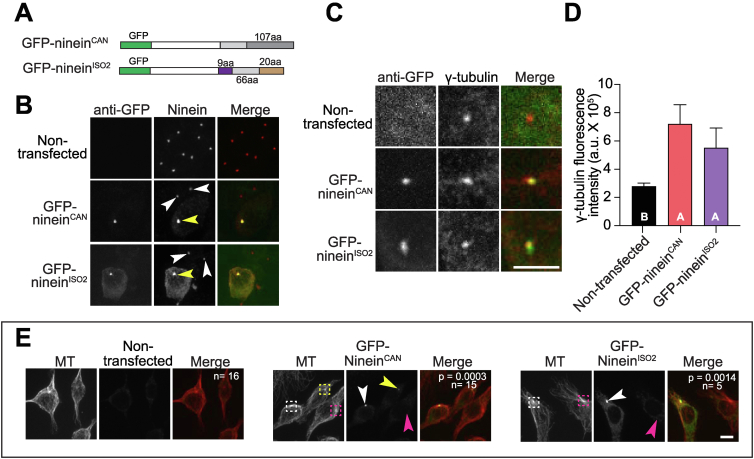


The activity of ninein^CAN^ has been studied in multiple cellular contexts including neurons and polarized epithelial cells (6, 7, 20). To the best of our knowledge, the expression, localization, and activity of ninein^ISO2^ remains uncharacterized. First, we sought to examine the relative expression of ninein^CAN^ and ninein^ISO2^ transcripts in RAW cells using reverse transcriptase quantitative PCR (RT-qPCR). To selectively amplify ninein^CAN^ and ninein^ISO2^, we used exonic primers where at least one primer in each pair binds to unique C-terminus regions in each isoform. A total ninein transcript (ninein^TOT^) was amplified using primer pairs that bind to conserved amino (N)-terminus sequences present in both variants (Fig. 2*A*). Measurements of the amounts of ninein^CAN^ and ninein^ISO2^ relative to ninein^TOT^ showed that ninein^CAN^ constitutes 85.17% ± 0.05 of the total ninein pool (Fig. 2*B*) a level approximately ∼9-fold more than ninein^ISO2^. To investigate the cellular localization of ninein^ISO2^, we generated N-terminally GFP-tagged ninein^ISO2^ using HiFi-assembly. RAW cells were transiently transfected with GFP- ninein^ISO2^ construct and compared to non-transfected cells and cells transfected with GFP- ninein^CAN 20^. Cells were then fixed and immunostained using an anti-GFP antibody. We found that, similar to endogenous ninein and GFP-ninein^CAN^, ectopic expression of GFP-ninein^ISO2^ localized as a central focus reminiscent of the centrosome (Fig. S3, *A* and *B*). We found that both GFP- ninein^ISO2^ and GFP- ninein^CAN^ enhanced the recruitment of γ-tubulin to the centrosome (Fig. S3, *C* and *D*) and increased MT polymerization from these areas (Fig. S3*E*). These co-localization analyses revealed that ninein^ISO2^ has similar characteristics to the well-studied isoform ninein^CAN^, despite differences in their primary sequences and predicted 3D structures.

To examine the function(s) of ninein^CAN^ and ninein^ISO2^ isoforms, we employed siRNA depletion strategies targeting unique C-terminus regions in the ninein^CAN^ or ninein^ISO2^ variant (Fig. 2*A*). Cells were also transfected with SMARTpool siRNA that targeted all ninein isoforms, ninein^TOT^, as well as non-targeting control siRNA, as described (26). To determine the specificity of siRNA-mediated depletion of ninein^CAN^ and ninein^ISO2^, we extracted RNA and generated total cDNA of RAW cells treated with ninein^TOT^-, ninein^CAN^-, ninein^ISO2^-, and non-targeting control siRNA. Using RT-qPCR, we measured the expression levels of ninein^CAN^ and ninein^ISO2^ transcripts using the isoform-specific primers. We found that amplification with ninein^CAN^- specific primers (qF2/qR2; Fig. 2*A*) yielded significantly less product in ninein^CAN^- and ninein^TOT^-depleted cells (Fig. 2*C*) compared to non-targeted cells, indicating an effective population-wide depletion of the ninein^CAN^ isoform. On the other hand, using primers to detect ninein^ISO2^ transcript (qF3/qR3; Fig. 2*A*) showed a substantial decrease in ninein^ISO2^ transcript in ninein^TOT^-depleted cells and to a lesser extent in ninein^ISO2^-depleted cells, compared to control cells treated with non-targeting siRNA (Fig. 2*C*). Importantly, ninein^CAN^ siRNA treatment did not alter ninein^ISO2^ transcript levels, and ninein^ISO2^ siRNA had no effect on ninein^CAN^ transcript levels (Fig. 2*C*), confirming the specificity of the siRNA targeting. Next, we examine the efficiency of ninein isoform depletion at the protein level using western blotting (Fig. 2*D*) and at the single-cell level with immunostaining (Fig. 2, *E* and *F*). Since there are no available isoform-specific ninein antibodies, we depleted cells of each isoform and then probed cells with an anti-ninein antibody against the common N-terminus epitope. Immunoblotting with the pan ninein antibody showed reduced ninein expression in the lysates of ninein^TOT^, ninein^CAN^, and ninein^ISO2^-depleted cells compared to control cells (Fig. 2*D*). The targeting of isoforms was also assessed by immunostaining of ninein-depleted cells (Fig. 2*E*). In RAW cells treated with ninein^CAN^ siRNA, we observed a drastic reduction of total ninein signal at the centrosome, compared to ninein^ISO2^ siRNA-treated cells. Measurements of ninein fluorescence intensity at the centrosome in ninein^TOT^-depleted cells resulted in an 84.54% reduction in fluorescence levels relative to the control levels (Fig. 2*F*). In ninein^ISO2^-depleted cells, ninein fluorescence intensity at the centrosome was reduced to about 36.0% relative to the control levels, compared to 64.9% in the ninein^CAN^-depleted cells (Fig. 2*F*). Taken together, our analysis shows that ninein^CAN^ is the predominant isoform in macrophages at both the mRNA and protein levels.

### Ninein^CAN^ and ninein^ISO2^ isoforms are both required for centrosome integrity and MT nucleation in macrophages

**Figure 3:**
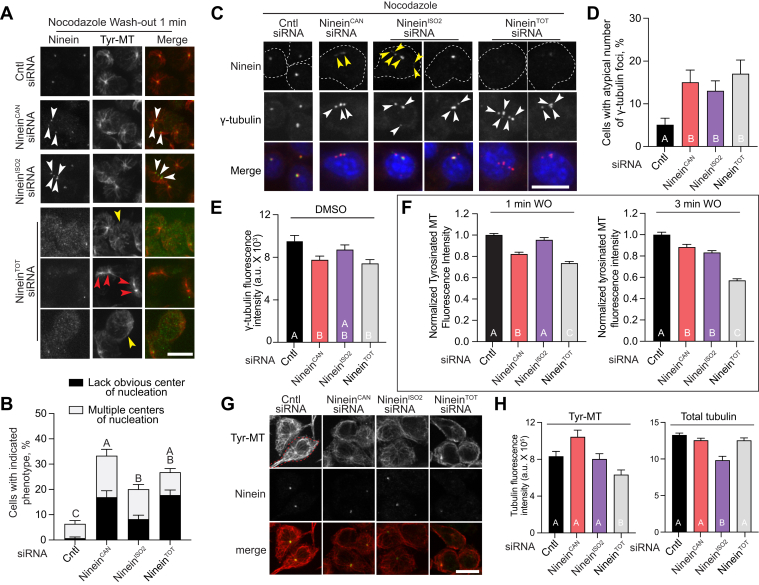
**Ninein^CAN^ and ninein^ISO2^ isoforms are required for an intact centrosome and MT nucleation.***A*, representative immunofluorescence images of cells treated with ninein^TOT^, ninein^CAN^, ninein^ISO2^, or control siRNA, followed by 30 min of nocodazole (NOC) treatment, washout (WO), and a 1 min MT regrowth period. Cells were immunostained for α-tyrosinated (Tyr) MTs and ninein. *White* arrowheads indicate the presence of multiple cytosolic foci enriched for both ninein and Tyr-MTs, while *yellow* arrowheads point to cells with unfocused nucleation sites where MTs originate from undefined locations and *red* arrowheads show Tyr-MTs enrichment at sites lacking ninein. *B*, percentage of cells displaying multiple or unknown nucleation sites after 1 and 3 min of MT regrowth following NOC washout in cells treated with the indicated siRNAs (n ≥ 213). Statistical significance is shown for combined phenotypes of unknown or multiple nucleation sites. *C*, confocal images showing an atypical number of centrosomes in cells treated with the indicated siRNAs, immunostained for γ-tubulin and ninein. *White* and *yellow arrowheads* indicate sites enriched for γ-tubulin and ninein, respectively. The *dashed line* shows the cells' outline. *D*, percentage of cells displaying an atypical number of γ-tubulin foci. Atypical is defined as cells with ≥ three γ-tubulin foci. Cells were treated with ninein^TOT^, ninein^CAN^, ninein^ISO2^, or control siRNA, followed by a 30-min NOC treatment (n ≥ 135). *E*, Quantification of γ-tubulin signal at the centrosome in cells treated with ninein^TOT^, ninein^CAN^, ninein^ISO2^, or control siRNA (n ≥ 106). *F*, quantification of Tyr-MTs fluorescence intensity in cells treated with the indicated siRNAs, followed by NOC treatment for 30 min, WO, and MT regrowth for 1 and 3 min. Fluorescence intensities are plotted relative to the geometric mean of the control siRNA condition for each replicate. *G*, representative confocal images of cells treated with DMSO under the indicated siRNA conditions, fixed, and immunostained for Tyr-MTs. *H*, Plot showing integrated density of Tyr-MTs (n ≥ 74) and total α-MTs (n ≥ 60). Letters denote significant differences between treatments (*p* < 0.05). The *p*-values were calculated using one-way ANOVA followed by Tukey's multiple comparisons test or the Kruskal-Wallis test, based on data from three independent experiments (*A–F*) or two experiments (*H*). Error bars represent standard error of proportion (SEP) in (*B* and *D*) and SEM in (*E*, *F*, and *H*). Scale bars = 10 μm.

To examine the contribution of each isoform to MT nucleation and MT network organization, we performed MT regrowth assays following MT depolymerization with nocodazole (36). Following treatment of RAW cells with isoform-specific siRNA, total ninein-targeting, or non-targeting control siRNA, cells were treated with nocodazole for 30 min, washed extensively, and then MTs were allowed to regrow for 1 and 3 min. Fixed cells were immunostained for ninein and tyrosinated (Tyr)-α-tubulin to label newly formed, dynamic MTs (37). In contrast to control cells, where Tyr-MTs emanated from a single site, at the centrosome, image analysis of MTs in cells treated with ninein^CAN^, ninein^ISO2^, and total ninein-targeted siRNA revealed that Tyr-MTs emanating from multiple sites including ninein-positive and ninein-negative sites (Fig. 3*A*). Furthermore, in ninein^CAN^, ninein^ISO2^, and ninein^TOT^-targeted siRNA-treated cells, we observed cells with Tyr-MTs nucleating from unidentified sites in the cytosol, leading to an overall unfocused and disorganized MT arrangement (Fig. 3*A*). Quantification of cells with defective MT organization showed that ninein^CAN^, ninein^ISO2^, and total ninein-depleted cells contained a significantly higher percentage of cells with disorganized Tyr-MT arrangements or contained multiple ectopic MT-nucleating sites, compared to control cells (Fig. 3*B*). We next examined whether the observed ectopic ninein foci under nocodazole treatment and isoform-depletion conditions contained γ-tubulin, an essential component of MT nucleation at the centrosome (38). Two-color analysis of ninein and γ-tubulin showed that not all ninein ectopic foci colocalized with γ-tubulin, and multiple ectopic γ-tubulin foci were observed in the absence of ninein in ninein^TOT^-depleted cells (Fig. 3*C*). Quantification of cells with more than two γ-tubulin foci showed that ninein^CAN^, ninein^ISO2^, and ninein^TOT^-depleted cells contained a significantly higher percentage of cells with an atypical number of γ-tubulin foci (Fig. 3*D*). To assess the relative contributions of ninein isoforms to the centrosomal localization of γ-tubulin, we examined the γ-tubulin signal in RAW cells treated with isoform-specific siRNA, total ninein-targeting, or non-targeting control siRNA-treated cells. We found that intensity measurements of γ-tubulin in ninein^CAN^ -depleted cells, but not ninein^ISO2^-depleted cells, were similar to the ninein^TOT^ conditions (Fig. 3*E*), suggesting that the ninein^CAN^ isoform is a stronger contributor to the recruitment of γ-tubulin to the interphase centrosome, potentially because of its higher prevalence. Collectively, our analyses suggest that both ninein isoforms are essential for the assembly of a single, cohesive MT organizing center in macrophages.

To assess the relative contribution(s) of ninein^CAN^ and ninein^ISO2^ in MT nucleation at the centrosome, we measured the density of MT arrays in siRNA-treated cells that contained a single, intact MT nucleation site to avoid measurement inconsistencies in cells with multiple ninein/γ-tubulin foci (Fig. 3, *A*–*D*). Measurements of Tyr-MTs signal intensity at MT nucleation sites revealed that the depletion of ninein^TOT^ induced a significant reduction in Tyr-MTs signal compared to control cells. This reduction in Tyr-MTs levels in ninein^TOT^-depleted cells persisted after 1 min and 3 min of nocodazole-washout (Fig. 3*F*). Measurements of centrosome-derived Tyr-MTs levels in ninein^CAN^ and ninein^ISO2^-depleted cells following 1 and 3 min of nocodazole-washout were significantly enhanced compared to the ninein^TOT^ conditions (Fig. 3*F*). Overall, our analyses suggest that, beyond their structural role in forming a sole MT nucleation site (Fig. 3, *A*–*D*), both isoforms are also required for normal MT growth at the centrosome. We next analyzed the potential role of ninein in regulating MT density in fully assembled MT networks (without nocodazole treatment) under our ninein total and isoform-specific knockdown conditions. Quantification of whole cell levels of Tyr-MTs and total MTs (Fig. 3, *G* and *H*) were variable among biological replicas and inconclusive (see Discussion section): we observed reduced Tyr-MT levels in ninein^TOT^ but not isoform-specific knockdown cells *versus* reduced total MTs in cells lacking ninein^ISO2^ but not ninein^TOT^ or ninein^CAN^ (Fig. 3, *G* and *H*). Collectively, our analysis suggests that both ninein isoforms are required for efficient MT nucleation from a focused nucleation site in macrophages.

### Both ninein isoforms are needed for macrophage proliferation with ninein^CAN^ isoform playing an essential role in duplicated centrosome separation

**Figure 4:**
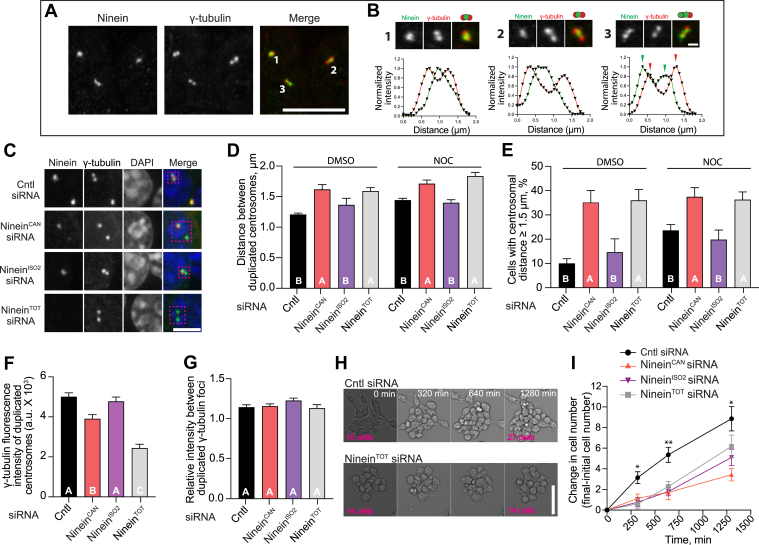
**Ninein^CAN^, but not Ninein^ISO2^, is required as a centrosome linker.***A*, representative confocal images of control cells treated with DMSO, fixed, and immunostained with γ-tubulin and ninein. *B*, Zoomed-in views of centrosomes from (*A*), highlighting the predominant spatial distributions of ninein and γ-tubulin signals. A representative normalized intensity profile of ninein and γ-tubulin signals along the duplicated centrosome is shown. *C*, representative confocal images of cells treated with the indicated siRNAs, showing duplicated centrosomes immunostained for γ-tubulin and ninein. Dashed boxes highlight duplicated centrosomes. *D*, measurements of the distances between duplicated centrosomes in cells treated with the indicated siRNAs and either DMSO (n ≥ 40) or NOC (n ≥ 101) for 30 min. Cells were fixed and immunostained with γ-tubulin and ninein antibodies. *E*, quantification of the percentage of duplicated centrosomes separated by ≥ 1.5 μm in cells treated with the indicated siRNAs and either under DMSO (n ≥ 40) or NOC (n ≥ 101) conditions. *F*, quantification of γ-tubulin enrichment at the centrosome in cells treated with ninein^TOT^, ninein^CAN^, ninein^ISO2^, or control siRNA (n ≥ 22). *G*, Plot showing the ratio of γ-tubulin intensity (bright/dim foci) in duplicated centrosomes (n ≥ 11). *H*, brightfield images showing cells treated with ninein^TOT^ or control siRNA. Cells were imaged for 22 h post a second dose of siRNA treatment. *I*, quantification of changes in cell number overtime. RAW cells were treated with indicated siRNA, followed by live imaging for 22 h following the second dose of siRNA treatment with 20 min intervals (n ≥ 313 cells examined across four different ROI in two replicates per condition). Significant differences between treatments are indicated by letters (*p* < 0.05). *p*-values were calculated using one-way ANOVA (*D–G*) and two-way ANOVA (*I*) followed by Tukey's multiple comparisons test or the Kruskal-Wallis test. Error bars represent the SEM in (*D*, *F*, *G*, and *I*) and SEP in (*E*). Scale bars = 10 μm (*A*), 1 μm (*B*), 5 μm (*C*), and 100 μm (*H*).

Ninein was recently implicated in duplicated centrosome cohesion, where depletion of ninein led to premature separation of centrosomes and a delay in nuclear envelope rupture in prophase cells (21). We sought to examine the potential involvement of ninein isoforms in regulating centrosome cohesion. First, we examined the spatial relationship between ninein and duplicated centrosomes in control macrophages. In control cells, ninein was found either as: a single focus between duplicated centrosomes, colocalized with only one of the duplicated centrosomes, or as two distinct foci that partially overlapped with the duplicated centrosome (Fig. 4, *A* and *B*). Next, we measured the distance between duplicated centrosomes marked by γ-tubulin in ninein^TOT^-, ninein^CAN^-, or ninein^ISO2^-depleted cells, compared to control siRNA-treated cells (Fig. 4, *C*–*E*). To exclude nascent bipolar spindles, measurements were limited to DAPI-stained cells showing intact nuclear envelopes with no condensed chromosomes. Distance measurements revealed that ninein^TOT^ depletion had longer distances between duplicated centrosomes, compared to control cells (Fig. 4, *C* and *D*), similar to observations in RPE1 cells (21). Furthermore, we found that the depletion of ninein^CAN^, but not ninein^ISO2^, resulted in larger distances between the duplicated centrosomes (Fig. 4*D*). We also quantified the percentage of cells with separated centrosomes using 1.5 μm as a minimal criterium for separated centrosomes. We found that the depletion of ninein^CAN^, but not ninein^ISO2^, resulted in an enhanced percentage of cells with separated centrosomes (Fig. 4*E*). Interestingly, the depletion of ninein^CAN^ recapitulated the defects observed after total ninein depletion (Fig. 4, *C*–*E*), suggesting a significant role for this isoform in centrosome cohesion. Duplicated centrosomes are held together by complementary mechanisms; cohesive linker proteins forming a physical tether between the proximal ends of the centrosomes as well as interdigitating MTs from each centrosome creating mechanical linkage by overlapping and interacting with motor proteins (21, 39, 40, 41). To test whether ninein^ISO2^ participation in centrosome cohesion relies on MTs, we used the MT-depolymerizing drug nocodazole. Depletion of ninein^ISO2^ and nocodazole treatment resulted in similar centrosome distances to control cells (Fig. 4, *D* and *E*), suggesting that ninein^ISO2^ is a dispensable component in linking duplicated centrosomes.

Next, we asked whether the depletion of ninein isoforms alters the recruitment of γ-tubulin to the duplicated centrosomes in cells treated with isoform-specific siRNA, total ninein-targeting, or non-targeting control siRNA. Measurements of γ-tubulin signal intensity revealed that the depletion of ninein^TOT^ induced a significant reduction in the level of γ-tubulin in duplicated centrosomes, compared to control cells (Fig. 4*F*). We also found that the depletion of each ninein isoform enhanced γ-tubulin recruitment to the duplicated centrosomes compared to ninein^TOT^-depleted cells (Fig. 4*F*), suggesting their contribution to γ-tubulin recruitment. Intriguingly, despite the asymmetric localization of ninein foci relative to the duplicated centrosomes (Fig. 4, *A* and *B*), the signal intensity ratio between the duplicated γ-tubulin foci remained balanced (Fig. 4*G*). Collectively, our analysis suggests that ninein^CAN^ helps bring duplicated centrosomes together as well as promotes γ-tubulin recruitment. Given the known role of ninein in cell cycle progression and proliferation (13, 14, 22), we next assayed macrophage proliferation under our ninein siRNA conditions. Using an automated brightfield live cell imaging microscope, we tracked the proliferation of RAW cells treated with either control-, ninein^CAN^-, ninein^ISO2^-, or ninein^TOT^-targeting siRNA. RAW cells were imaged for over 22 h post-siRNA treatment. Tracking of the cell number changes over time revealed that ninein^TOT^-depleted cells had reduced cell numbers compared to the control cells (Fig. 4, *H* and *I*), consistent with previous observations of neuronal apical progenitor cells following ninein-depletion (13). Similar to cells lacking total ninein, we found that both ninein^CAN^- and ninein^ISO2^-depleted cells showed reduced cell numbers compared to control cells (Fig. 4, *H* and *I*), suggesting that both isoforms are required for normal macrophage proliferation.

### Ninein isoforms are required for dynein recruitment to the centrosome but only ninein^CAN^ contributes to Golgi positioning in macrophages

**Figure 5:**
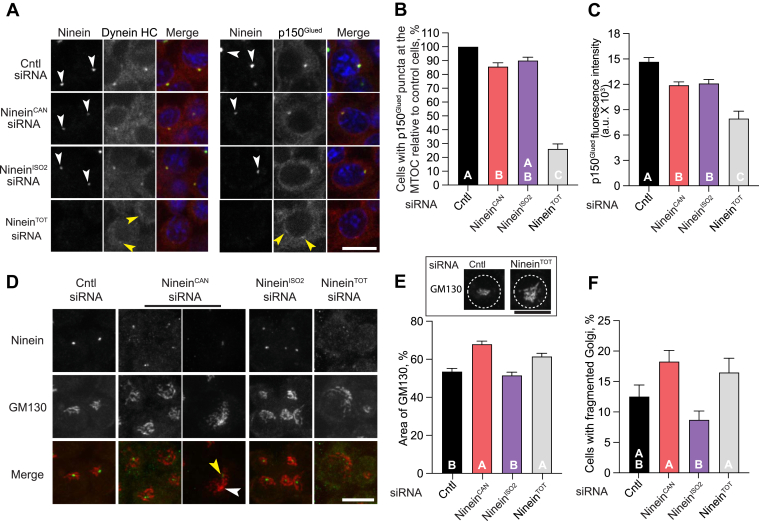
**Ninein isoforms are required for dynein recruitment to the centrosome, but only ninein^CAN^ participates in Golgi distribution.***A*, representative confocal slices of RAW cells treated with ninein^TOT^, ninein^CAN^, ninein^ISO2^, or control siRNA, fixed, and immunostained for dynein heavy chain (HC) and the dynactin subunit p150^Glued^. *White* arrowheads indicate ninein localization at the centrosome, while *yellow* arrowheads highlight ectopic p150^Glued^ localization. *B*, quantification of the percentage of cells with centrosomal p150^Glued^ localization relative to the control siRNA condition (n ≥ 126). *C*, plot showing the integrated density of centrosomal p150^Glued^ relative to background signal (n ≥ 25). *D*, Maximum intensity projections of RAW cells treated with the indicated siRNAs. Cells were fixed and immunostained for ninein and GM130. *White* arrowheads indicate Golgi distributed at a distance from the remaining ninein signal at the centrosome (*yellow arrowheads*). *E*, representative images showing Golgi spread in ninein-depleted cells compared to control cells. Quantification of the area of GM130 signal, which increases upon fragmentation, in RAW cells treated with the indicated siRNAs (n ≥ 75). (F) Quantification of the percentage of cells with fragmented Golgi in cells treated with the indicated siRNAs (n ≥ 249). Letters indicate significant differences between treatments (*p* < 0.05). The *p*-values were calculated using one-way ANOVA followed by Tukey's multiple comparisons test or the Kruskal-Wallis test from two experiments (*A–C*) and three experiments (*D–F*). Error bars represent SEP in (*B* and *F*) and SEM in (*C* and *E*). Scale bars = 10 μm.

Ninein has been shown to activate dynein-dynactin processive movement in single-molecule motility assays *in vitro* (25), and depletion of ninein disrupts dynein recruitment to the phagocytic cup (26). We examined dynein/dynactin localization in resting macrophages and found that, as expected (26), dynein heavy chain (HC) and the dynactin subunit p150^Glued^ are both localized to the centrosome (Fig. 5*A*). In ninein^TOT^-depleted cells, we observed a dramatic reduction in the accumulation of dynein and dynactin at the centrosome (Fig. 5, *A* and *B*). Furthermore, image analysis of the remaining p150^Glued^ foci revealed significantly reduced intensity levels compared to the control cells (Fig. 5*C*). Comparison of total ninein depletion and isoform-specific depletion showed that ninein^CAN^ and ninein^ISO2^-depleted cells, retained higher dynactin localization than ninein^TOT^-depleted cells; however, this localization was significantly lower than in control levels (Fig. 5, *B* and *C*). This suggests that both isoforms are needed for efficient recruitment of dynein/dynactin to the centrosome. Dynein/dynactin activity holds the Golgi complex adjacent to the centrosome, and the inhibition of dynein or its adaptors results in fragmentation and dispersion of the Golgi ribbon (3, 42, 43, 44). To test the functional consequences of ninein isoform loss on dynein functions, we examined whether the depletion of ninein^CAN^- or ninein^ISO2^-depleted cells affected Golgi morphology and distribution in RAW cells. Following treatment of cells with isoform-specific siRNA, cells were fixed and immunostained for ninein and GM130, an abundant cis-Golgi resident protein. Measurements of the Golgi network's spread revealed a larger distribution area in cells depleted for ninein^TOT^ and ninein^CAN^, but not in cells depleted for ninein^ISO2^ (Fig. 5, *D* and *E*). We also quantified the percentage of cells with fragmented Golgi and found an enhanced percentage of cells with fragmented Golgi in ninein^TOT^ and ninein^CAN^, but not ninein^ISO2^-depleted cells (Fig. 5*F*). These findings indicate that although both isoforms are needed for dynein/dynactin recruitment to the centrosome, only ninein^CAN^ plays an important role in Golgi morphology and distribution.

### Ninein^ISO2^ plays a major role in F-actin recruitment and particle internalization during FcγR-mediated phagocytosis

**Figure 6:**
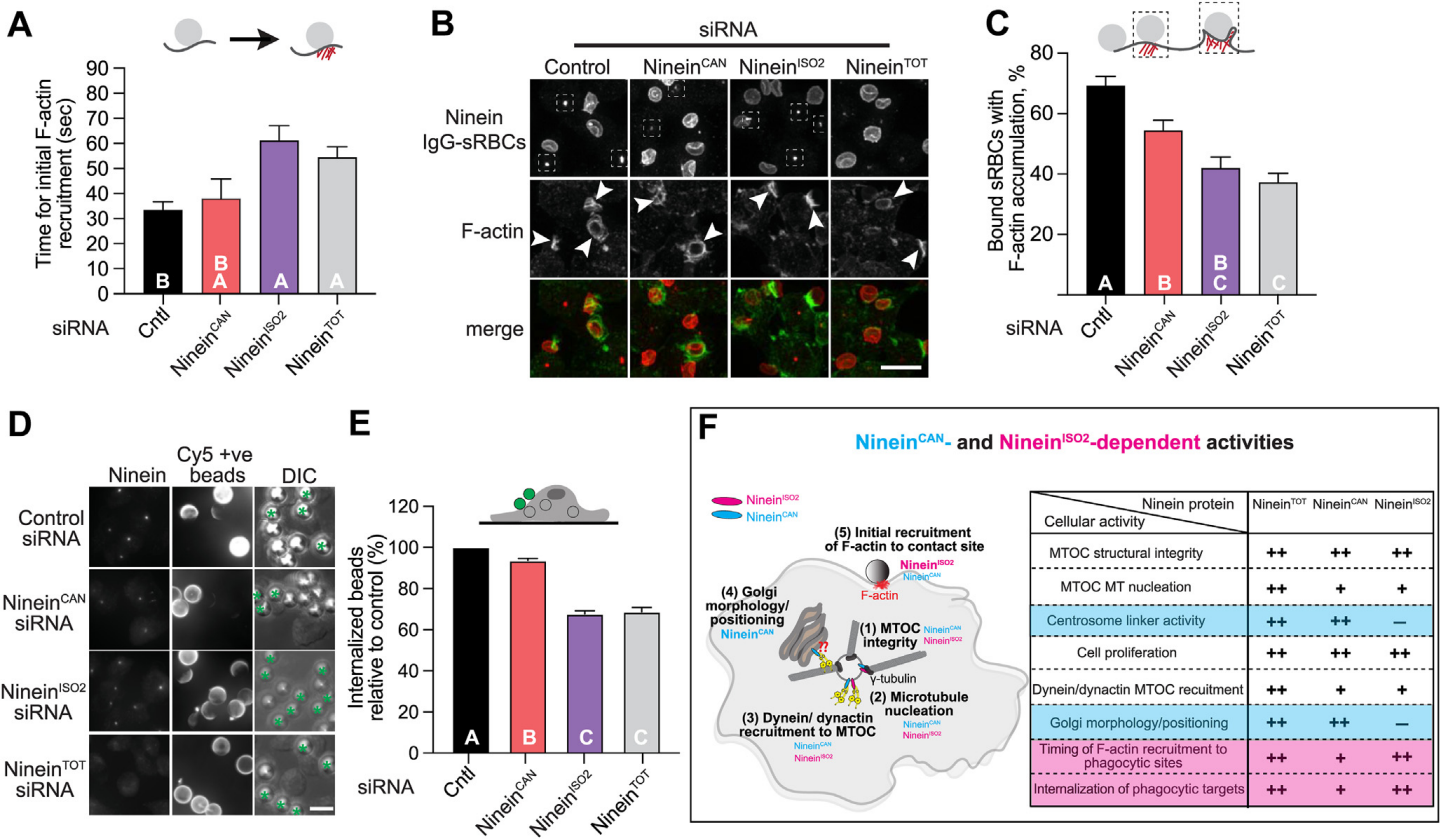
**Ninein^ISO2^ plays a major role in facilitating F-actin recruitment and particle internalization in macrophages.***A*, quantification of the average duration (in seconds) from the binding of a particle to the cell surface (t = 0 s) to the initial F-actin recruitment at the contact site (n ≥ 12) in RAW cells treated with ninein^TOT^, ninein^CAN^, ninein^ISO2^, or control siRNA. *B*, representative confocal images showing the binding of IgG-sRBCs to the cell surface of RAW cells treated with the indicated siRNAs. *Arrows* indicate F-actin enrichment at the IgG-sRBC-contact site on the macrophage surface, with a box highlighting the location of ninein at the centrosome. *C*, quantification of the percentage of bound IgG-sRBCs with F-actin polymerization at the contact site (n ≥ 181) under the indicated conditions. *D*, representative epifluorescence images of RAW cells treated with the indicated siRNAs. Cells were challenged with 6.92 μm IgG-coated beads for an 8 min pulse, washed, and chased for 12 min to assess internalization efficiency. Cells were fixed, immunostained for external beads, permeabilized, and immunostained for ninein. Green asterisks indicate bound and/or incompletely internalized particles labeled with Cy5-anti-IgG. *E*, percentage of internalized beads (number of completely internalized beads/total bound plus internalized beads) in cells treated with the indicated siRNAs (n ≥ 1148 beads). *p*-values were calculated using one-way ANOVA followed by Tukey's multiple comparisons test or Kruskal-Wallis test from two experiments (*B* and *C*) and three experiments (*A*, *D*, and *E*). Error bars represent SEM (*A*) and SEP (*C* and *E*). Scale bars = 10 μm. *F*, proposed roles for ninein^CAN^ and ninein^ISO2^ isoforms. Both ninein isoforms localize at the centrosome to enhance dynein-dynactin complex recruitment to the centrosome. The presence of ninein^CAN^ is important for the Golgi morphology and positioning close to the centrosome, likely through interactions with the MT cytoskeleton. After particle binding, ninein^ISO2^ plays a primary role in the initial F-actin recruitment to the particle contact site to promote timely cup formation and efficient internalization during FcγR-mediated phagocytosis in macrophages.

Finally, we sought to determine the requirement of ninein isoforms in the critical function of phagocytosis in macrophages. Our previous work demonstrated that ninein is required for the timely accumulation of F-actin at the site of phagocytosis and facilitates the retrograde movement of nascent phagosomes (26). We treated resting RAW cells with siRNA to deplete total or individual ninein isoforms, then challenged the cells with IgG-opsonized sheep RBCs (IgG-sRBCs). Using RAW cells stably expressing the fluorescent LifeAct construct (LifeAct-RFP), we tracked F-actin dynamics at the phagocytic membrane using live cell confocal microscopy. In control cells, we observed a robust enrichment of the F-actin fluorescence signal at the particle-contact site following contact with IgG-sRBCs (Fig. 6*A*). In contrast, ninein^TOT^- and ninein^ISO2^-depleted cells showed longer time intervals between IgG-sRBC binding and the appearance of the LifeAct fluorescence signal at the particle attachment sites, compared to control cells (Fig. 6*A*). To confirm this observation, we used single time-point analysis challenging ninein^CAN^-, ninein^ISO2^-, and ninein^TOT^-depleted cells with IgG-sRBCs for 4 min, a time sufficient for particle binding, but not complete internalization, before cells were fixed and immunostained for ninein and endogenous actin. In the absence of ninein^TOT^ and ninein^ISO2^, a smaller percentage of bound particles were able to elicit F-actin recruitment to the site of contact, compared to bound IgG-sRBCs in control cells (Fig. 6, *B* and *C*). This analysis also revealed that knockdown of the ninein^CAN^ isoform impaired F-actin recruitment to the particle-contact site, albeit to a lesser degree than ninein^ISO2^ knockdown (Fig. 6, *B* and *C*). Next, we assessed particle internalization efficiency, by distinguishing engulfed beads from bound beads by staining cells with anti-IgG-Cy5 before cell permeabilization. We quantified the percentage of internalized beads relative to total number of beads (number of internalized beads/sums of bound and internalized beads). We found a significant decrease in the percentage of internalized IgG-beads in ninein^TOT^-depleted cells compared to control cells (Fig. 6, *D* and *E*). Consistent with observations in Figure 6*C*, ninein^CAN^ isoform knockdown resulted in a slight reduction in internalization, while ninein^ISO2^ knockdown showed similar reductions compared to total ninein depletion. Taken together, our analysis suggests that ninein^ISO2^ is the prominent isoform in regulating F-actin recruitment and internalization during FcγR-mediated phagocytosis in macrophages.

## Discussion

### Ninein isoform generation in mouse macrophages likely uses several alternative splicing mechanisms

In mammalian cells, isoform diversity is generated through mechanisms like alternative splicing and the use of alternative transcription start and stop sites (45, 46, 47, 48). Our analysis of ninein in mouse macrophages pulled out two main isoforms and some insight into their complex generation. We found that ninein^ISO2^ is likely produced through a combination of two alternative splicing events, utilizing distinct inclusion patterns that may contribute to combinatorial diversity (49). The usage of a 5′ alternative splicing donor site at the exon-intron boundary generates the 9 aa motifs, while the usage of an alternative 3′-terminal acceptor site results in the formation of the last variable 20 aa C-terminus and an alternative 3′UTR. Although the selection of alternative 5′ or 3′ splice sites at exon-intron junctions is a less common mechanism, these events account for at least 25% of known alternative splicing occurrences (46, 47). Previous studies have revealed that alternative splicing *via* exon skipping is used to generate the mouse isoform ninein^Neuro^ (15, 28, 29). Specifically, exon 18 is included in neuronal precursor cells but is skipped in neurons during differentiation (15, 28, 29). Exon skipping is considered the most prevalent mechanism of alternative splicing in mammalian cells (47, 50). Recently, RNA sequencing of brain tissue of C57BL/6J and DBA/2J mice identified additional exon-skipping events in ninein transcripts between the two strains, including three novel splicing events in the 3′end in addition to multiple SNPs differentially expressed between the two mice strains that are correlated with distinct physiological responses (28).

The evolutionary conservation of mouse ninein^ISO2^ with its human orthologues, particularly the 9 aa motif sequence, argues for its functional relevance given that the two species are separated by 75 to 110 million years (51). We found a strong resemblance of the 20 aa in the C-terminus of ninein^ISO2^ with human ninein isoform 1, showing a 90% identity and 0% gap. Ninein isoform 1 is expressed in different human tissues and localizes to the centrosome (22, 30). Ninein^ISO2^, ninein^ISO3^, and ninein^Neuro^ contain alternative 3′ UTR that potentially like other transcripts impacts post-transcriptional control of gene expression and influences mRNA stability (52, 53) and translation (54). Consistent with this notion of differential ninein isoform expression, we observed a much higher abundance of ninein^CAN^ in mouse macrophages compared to ninein^ISO2^. Ninein^CAN^ isoform is generated through constitutive splicing where all introns are removed, and all 30 exons are ligated in the order that they appear in the ninein gene. Thus, the divergent C-terminus and 3′UTR between the two isoforms may reflect differences in the regulation of ninein (28) at transcriptional or post-transcriptional levels.

### Potential presence of additional ninein isoforms in macrophages

While our analysis confirms the presence of ninein^CAN^ and ninein^ISO2^ isoforms in RAW macrophages, we cannot rule out the existence of additional isoforms in mouse macrophages, particularly if they are expressed at a low level. This could be a limitation of the PCR-based approaches where the low-copy number transcript is outcompeted by dominant isoforms and/or by Sanger sequencing approach limitations in differentiating between two alleles that are present in substantially unequal proportions. Additionally, the potential presence of multiple ninein isoforms with sequence homology would result in off-target effects of siRNA. For instance, phenotypes attributed to ninein^CAN^ depletion could be influenced by concurrent depletion of multiple isoforms with siRNA-targeted sequence homology such as ninein^ISO4^ and phenotypes observed upon ninein^ISO2^ depletion might reflect contributions from ninein^ISO4^ or ninein^Neuro^. The close similarity among ninein isoforms, combined with the limitations of optimal primer length in RT-qPCR, could also affect the accurate quantification of ninein transcripts. If additional isoforms are present in macrophages, then the qF2/R2 primers used for ninein^CAN^ detection may also amplify ninein^ISO4^, while the qF3/R3 primer pair could detect ninein^ISO4^ alongside ninein^ISO2^.

### The interplay between ninein isoform diversity and centrosome structure and function in mouse macrophages

Ninein isoforms exhibit variations primarily, but not exclusively, in the C-terminal region (15, 22, 28, 30). These isoforms are co-expressed within the same tissue (30) or show differential expression, in a cell type-specific manner (15). Among the identified ninein isoforms, loss of centrosomal localization is associated with isoform diversity in humans and mice. For instance, human ninein isoforms 1, 2, and 5 exhibit centrosomal localization, while isoform 6 is non-centrosomal (22, 30). In mice, ninein^ISO2^ and ninein^CAN^ localize to the centrosome, whereas ninein^Neuro^ appears cytosolic (15). The switch to non-centrosomal isoforms is potentially correlated with the attenuation of MT nucleation at the centrosome and the establishment of non-centrosomal MT nucleating sites, as observed during the differentiation of neural progenitor cells into neurons (15). In mouse macrophages, we did not observe marked differences in the centrosomal localization of ninein^CAN^ and ninein^ISO2^ isoforms, despite the primary sequence variation at the C-terminus. The central region of ninein^Neuro^, but not the C-terminus, is responsible for centrosomal targeting *via* interactions with CEP170 and CEP250 (15). However, localization experiments using truncations of mouse ninein^CAN^ isoform showed that the C-terminus fragment, but not the central region, is required for centrosome targeting (20). Localization of human ninein is similarly inconclusive, where the C-terminus region was reported to be contributing (30) or dispensable (22) for centrosomal recruitment.

Through depletion strategies, we identified ninein^ISO2^ and ninein^CAN^ as key players in maintaining a single, intact, and functional centrosome. In the absence of either isoform, ectopic aggregates of ninein and γ-tubulin were observed under nocodazole treatment conditions. Overexpression of ninein^CAN^ (20) and central fragments of human ninein isoforms (22, 30) formed ectopic non-centrosomal sites containing γ-tubulin but without MT nucleating capacity. The role of ninein in promoting MT nucleation is controversial. While some reports have shown that total ninein depletion (21) or overexpression of dominant-negative C-terminus truncations of ninein^CAN^ (20) reduced γ-tubulin and MTs at the centrosome, other studies (9, 10, 13) showed that γ-tubulin levels at the centrosome were not affected by the reduction/mis-localization of endogenous ninein. Our findings here suggest that both ninein isoforms promote MT nucleation by recruiting γ-tubulin to centrosomal or experimentally-induced cytosolic foci. It is intriguing, that despite ninein^CAN^ being highly abundant compared to ninein^ISO2^, a similar potent impact on MT organization was observed when either isoform was depleted. Examining the contributions of total ninein and individual isoforms on steady-state MT organization and dynamics is challenging due to the complex effects of ninein depletion on MT organization. These effects range from a reduced MT network in some cells to others showing multiple ectopic nucleating sites. Consequently, cell-to-cell variability obscured a detailed understanding of the role of ninein and its isoforms on interphase MT organization.

Ninein homozygous null mutants in *Drosophila* exhibit chromosome segregation defects and detachment of centrosomes from mitotic spindles (55). In RPE1 cells with supernumerary centrosomes, ninein depletion was reported to lead to metaphase delay (21), impairing pseudo-bipolar spindle formation, and resulting in mis-segregation of chromosomes. Ninein activities are not limited to interphase, as overexpression of human ninein in HeLa cells inhibited cell growth and impaired entry into mitosis (22, 30). Although the molecular details underlying these defects are not completely clear, some of these phenotypes could be attributed to ninein's role as a cohesion linker between duplicated centrosomes (21). Duplicated centrosomes are held together by the major cohesive protein cNap1 at the proximal ends of the centrosomes as well as by kinesin-like protein KIF11 acting on interdigitating MTs emanated from each centrosome (21, 56, 57). Upon mitotic entry, separase cleaves these linkages (58, 59) to allow the two centrosomes to move further apart to form the spindle poles in a time-regulated manner. Ninein has been shown to colocalize with cNap1 (60) and its localization is dependent on the presence of cNap1 (21). Based on our protein isoform knockdown studies, ninein^CAN^ appears to be the sole isoform required for centrosome cohesion in mouse macrophages. The reason why ninein^CAN^, but not ninein^ISO2^, is required for centrosome cohesion remains elusive. One potential explanation is that the relatively high abundance of ninein^CAN^ facilitates cNap1 activity, which is known to accumulate at centrosomes at micromolar concentrations (41).

### Role of ninein isoforms in dynein/dynactin localization and activity in mouse macrophages

Our structural analysis revealed that changes in the ninein^ISO2^ primary sequence, compared to ninein^CAN^, still resulted in a characteristic elongated dimer that is typical of dynein adaptors (34, 61) and preserved interactions with dynein at the centrosome. In several model organisms, ninein has been shown to associate with the dynein/dynactin motor complex (25, 26, 27, 62). Ninein-dynein interactions depend on the N-terminus of ninein, as the fragment (1–693 aa) of human ninein was sufficient to induce dynein motility *in vitro* (25). In addition, analysis of the *Drosophila* Nin-RB isoform revealed that the N-terminus fragment (1–353 aa) is bound to the dynein light intermediate chain (63). These functional conservations across different species suggest that ninein is a key player in dynein recruitment.

Golgi positioning and morphology depend on MTs and the motor dynein (3, 64). In addition to MT organization and recruitment of dynein to the centrosome, it is plausible that ninein participates in organizing MTs on the Golgi membrane. For instance, a ninein-interactome BioID dataset in HEK293-T cells identified interactions with CEP350 (>30-fold enrichment) and CAMSAP1, 2, and 3 (>5–10 fold enrichment) relative to a BioID control (25). CAMSAP2 and CAMSAP3 participates in the organization of non-centrosomal MTs on the Golgi and Golgi morphology (65, 66). CEP350 stabilizes MTs at both the centrosome and Golgi, and its depletion or overexpression-induced Golgi fragmentation (67). Intriguingly, CAMSAP1, CAMSAP2, and CAMSAP3 were detected in ninein C-terminus BioID assays, but not in ninein N-terminal interactomes (25), potentially explaining our differential requirement for ninein^CAN^ *versus* ninein^ISO2^ in Golgi positioning in macrophages. This is also in agreement with dynein adaptors mediating cargo binding *via* their C-terminus, a structural feature revealed using high-resolution cryo-EM (34). These results warrant further investigation into how sequence-based/structural differences between ninein isoforms influence their form and function.

### Ninein^ISO2^ is critical for FcγR-mediated phagocytosis in mouse macrophages

Our previous work showed that total ninein depletion impaired efficient FcγR-mediated phagocytosis (26) and here we show that ninein^ISO2^ is the major contributor to this critical innate immune process. We speculate that the unique 9 aa motif in ninein^ISO2^ mediates interaction with key F-actin regulators driving this process. The unique 9 aa motif is present in all human ninein isoforms but absent in ninein^CAN^. Structural predictions further suggest that the primary sequence differences between these two isoforms have a significant effect on helix orientation and compactness, potentially influencing their overall binding affinities or function. Supporting this, a BioID interactome of human ninein isoform 5 identified several actin regulators including cortactin and IQGAP1 (25). Cortactin is a known activator of the Arp2/3 complex (68) and is required for phagocytosis in Sertoli cells (69). IQGAP1 acts as a scaffold for the actin nucleator Dia1 (70, 71), and its absence leads to reduced internalization of avidin-coated latex beads (72). Future work will directly explore binding partners of ninein^ISO2^, specifically the unique 9 aa motif, to confirm its involvement in F-actin remodeling during FcγR-mediated phagocytosis.

Overall, our comprehensive cellular analysis reveals the involvement of ninein isoforms in both centrosomal and non-centrosomal activities. In mouse macrophages, the two major ninein isoforms share and partition roles in MT organization, dynein recruitment, and cargo-specific activity, including Golgi positioning and morphology during interphase. Interestingly, the abundant ninein^CAN^ isoform has a distinct contribution to centrosome cohesion during cell division while ninein^ISO2^ predominates in F-actin-driven phagocytosis in macrophages. This work provides molecular insight into the growing literature on ninein isoform functions in specialized mammalian cell types.

## Experimental procedures

### Antibodies and reagents

The following primary antibodies were used for immunostaining analysis: mouse anti-α-tubulin (1:10,000; T9026, MilliporeSigma, Canada Co), mouse anti-tyrosinated (Tyr) α-tubulin (1:1000; T9028, MilliporeSigma), mouse anti-γ-tubulin (1:500, T5326, MilliporeSigma), rabbit anti-ninein (1:300, ABN1720; MilliporeSigma), mouse anti-pan actin (1:200, AAN02, Cytoskeleton Inc), mouse anti-β actin (1:1000, A5441, MilliporeSigma), mouse anti-dynein HC (1:100, sc-514579, Santa Cruz Biotechnology Inc.), mouse anti-dynactin p150^Glued^ (1:500, 610474, BD Biosciences), rat anti-GFP (1:300, 101536, Santa Cruz), mouse anti-GM130 (610823, BD Biosciences), human IgG (I4506, MilliporeSigma), mouse anti-GAPDH (1:1000, SAB1403850, MilliporeSigma), and DAPI (1:10,000, MilliporeSigma). Cy2, Cy3, and Cy5 donkey anti-rat, anti-mouse, anti-human, and anti-rabbit secondary antibodies (diluted 1:500) were purchased from Jackson ImmunoResearch Laboratories. Sheep red blood cells (sRBCs) were obtained from MP Biomedical (ICN55876, Solon, OH). Rabbit anti-sRBC IgG (ICN55806) was obtained from MP Biomedical. The 6.92 μm (PS06004) crosslinked polystyrene divinylbenzene beads were purchased from Bangs Laboratories. Plasmid expressing mouse GFP-Ninein^CAN^ was gifted by Michel Bornens (Addgene plasmid #73519), which we sequenced to confirm its isoform identity. The mammalian expression plasmid of N-terminus mScarlet (Addgene #85042) was a gift from Dorus Gadella. DAKO Fluorescence mounting media was purchased from Agilent Technologies Canada. Dimethylsulfoxide (DMSO, DMS666.50, Bioshop), nocodazole (MilliporeSigma), DMEM (319-005-CL), fetal bovine serum (FBS, 085-450), and phosphate-buffered saline (PBS, 311-010-CL) were purchased from Wisent Inc. (St-Bruno, QC). ON-TARGET plus mouse ninein SMARTpool siRNA (L-062136-01-0010), ON-TARGET plus non-targeting pool siRNA (D-001810-10-05), Ninein^CAN^ (sense: 5′ A.A.A.C.C.A.G.G.A.A.C.A.C.C.U.U.G.U.A.A.A.U.C.U.U.U 3′ and antisense 5′-P.A.G.A.U.U.U.A.C.A.A.G.G.U.G.U.U.C.C.U.G.G.U.U.U.U.U 3′), and Ninein^ISO2^ (sense: 5′ G.U.G.A.G.A.C.U.G.G.A.C.G.A.G.A.A.G.C.U.A.A.U.G.G.U.U 3′ and antisense 5′-P.C.C.A.U.U.A.G.C.U.U.C.U.C.G.U.C.C.A.G.U.C.U.C.A.C.U.U 3′) were purchased from Dharmacon.

### Cell culture and siRNA transfection

The RAW 264.7 (RAW) murine macrophage cell line was purchased from the American Type Culture Collection (ATCC). RAW cells were cultured and maintained in complete DMEM containing 10% heat-inactivated FBS at 37 °C in 5% CO_2_. To deplete ninein^TOT^ or specific ninein isoforms, 0.3 to 0.5 × 10^5^ or 0.2 × 10^6^ cells were seeded onto coverslips in 24-well or 6-well plates, respectively, for ∼24 h prior to siRNA treatment. Cells were transfected with ninein^TOT^, ninein^ISO2^, ninein^CAN^, or control siRNA at a concentration of 50 to 100 nM, with daily dosing for 48 h Transfection of siRNA was performed using Lipofectamine 3000 Transfection Reagent (L3000001, Thermo Fisher Scientific) according to the manufacturer's instructions. LifeAct-RFP-expressing cells were produced in our laboratory by Dr He Song Sun. All cell lines were free from *mycoplasma* contamination for all experiments.

### Isoform identification and sequencing

Multiple sequence alignments of mouse ninein transcripts and isoform protein sequences (Sequence accession numbers: AAS87211, NP_001074922, NP_001390761, NP_001273009, NP_032723, and UniProt Q61043-4) were generated using Multiple Sequence Comparison by Log-Expectation (MUSCLE) algorithm (73). For pairwise sequence alignments, the Needleman-Wunsch global alignment was performed. All sequence visualization and alignments were carried out using SnapGene (GSL Biotech) version 7.2 or earlier. Exons number and intron-exon boundaries were assigned based on Ensembl transcript Nin-211. Multiple protein sequence alignments of ninein isoforms were used to identify amino acid differences between the isoforms. In addition to lack of central exons and/or variable C-termini, the alignment identified six sites identified in ninein^CAN^ (NCBI AY515727/AAS87211) with the following residues: K675, E909, Y1084, Q1085, K1155, and P1327 that differ from ninein^NPC^ (NP_001074922), ninein^ISO2^, and all other ninein isoforms (ninein^ISO3^ and ninein^ISO4^) which contain the missense variants E675, K909, S1084, R1085, E1155, and A1327. Examination of these genetic variations in Ensembl and the Mouse Genomes Project among 18 laboratory mouse strains and a recent report (28) suggest they may result from differences in laboratory mouse strains' background. Notably, no record on variants was found at positions 675 and 1327, but the following variants were annotated: K909E (reference SNP: rs29149025), S1084Y (reference SNP: rs29159683), R1085Q (reference SNP: rs29192398), and E1155 K (reference SNP: rs32225358). These analyses suggest that ninein^CAN^ (NCBI Accession: AAS87211) and ninein^NPC^ (NCBI Accession: NP_001074922/UniProt: Q61043-3) represent the same transcript expressed across different mouse strains. Thus, we only included ninein^CAN^ transcripts, consistent with the genetic background for RAW macrophages, as a representative of this isoform in subsequent analysis. Phylogenetic trees were constructed using Molecular Evolutionary Genetics Analysis (MEGA, version 11.0) (74) incorporating ninein isoforms from mice and humans and one isoform of the paralog ninein-like protein (Nlp). Protein isoform sequences were aligned using MUSCLE algorithm with default settings, and maximum likelihood analysis was performed with default settings to estimate phylogenies.

Total RNA was extracted from untreated or siRNA-treated cells RAW cells from three to four independent passages using the PureLink RNA Mini Kit (12183025, Thermo Fisher Scientific) following the manufacturer's protocol. cDNA synthesis was performed using SuperScript III First-Strand Synthesis SuperMix (11752050, Thermo Fisher Scientific) or SuperScript IV First-Strand Synthesis SuperMix (18091050, Thermo Fisher Scientific) using 1 to 2 μg of total RNA. To identify different mouse ninein splice variants, cDNA was amplified in reaction mixtures containing Q5 High-Fidelity DNA Polymerase (M0491, New England Biolabs, Ontario) and primers listed in Table 1. Amplification products were separated on 1 to 2% agarose gel before cDNA bands with the expected size were excised and purified using the QIAquick Gel Extraction Kit (28704, Qiagen). Sanger sequencing was performed at the Hospital for Sick Children. Sequencing reads were aligned using SnapGene and compared to publicly available ninein transcript sequences in UniProt, NCBI, and Ensembl databases. For splice site prediction, the splice site predictor tool (31) in the Berkeley *Drosophila* Genome Project was utilized. The predictor was run for both 5′ and 3′ splice sites using a minimum score threshold of 0.40.

AlphaFold2 v2.3.2 (75, 76) *via* the COSMIC2 platform (77) was used to predict the structures of ninein^CAN^ and ninein^ISO2^ isoforms. Default AlphaFold2 settings were applied, including five predictions per model, use of the full AlphaFold2 database, and no relaxed model calculations. No protein structure or sequence template was inputted for predictions. The pLDDT plots generated by AlphaFold2 were visualized using ChimeraX (78, 79, 80). AlphaFold3 (81) through the AlphaFold Server was used to predict homodimers of mouse ninein^CAN^ and ninein^ISO2^. No additional ligands or ions were included in the models.

### Plasmid construction

To study the localization of ninein^ISO2^, a plasmid expressing eGFP-ninein^ISO2^ under the CMV promoter was constructed using the NEBuilder HiFi DNA Assembly Cloning Kit (E5520S, New England Biolabs). Compared to eGFP-ninein^CAN 20^, we built eGFP-ninein^ISO2^ to contain a 9 aa unique motif and 20 aa variable C-terminus. However, not consistent with the genetic background for RAW macrophages, we included six mouse genetic background-specific substitutions (E-K675, K-E909, S-Y1084, R-Q1085, E-K1155, A-P1327). Briefly, we generated seven fragments amplified with Q5 High-Fidelity DNA polymerase and mutagenic primers designed to incorporate substitutions in addition to 20-22mers of complementary flanking sequences built into primer ends to generate overlapping sequences between each consecutive fragment. To amplify Fragments 1 to 6, eGFP-ninein^CAN^ plasmid (20) was used as a cDNA template. Fragment 1 amplification (using F4/R4; 2793 bp product) contained a Kozak consensus sequence for protein translation initiation, eGFP, a linker (Ser-Gly-Leu-Arg-Ser), and a 2035 bp of ninein N-terminus sequence that incorporated (K-E^675^) modification. Fragment 2 (amplified using F5/R5; 722 bp product) was used to introduce E-K^909^ substitution. Fragment 3 (amplified using F6/R6; 548 bp product) was used to introduce Y-S^1084^ and Q-R^1085,^ while Fragment 4 (amplified using F7/R7; 230 bp) introduced K-E^1155^. Fragment 5 (amplified using F8/R8; 536 bp) contained the P-A^1327^ variant, while Fragment 6 (amplified using F9/R9;1876 bp product) was used to introduce the 27 bp insertion that corresponded to the 9 aa unique sequence. Fragment 7 (amplified using F10/R10; 647 bp product) was amplified from mouse total cDNA generated from the total RNA of RAW cells. Primers used to generate this fragment bind to the sequence corresponding to the 9 aa unique sequence and the 3′UTR in addition to 20-mers of complementary overlapping sequences to the backbone of the vector (Addgene# 85042).

Following PCR, the amplification products were separated on 1 to 2% agarose gel, excised, and purified using QIAquick Gel Extraction Kit. Using overlap extension PCR, we ligated Fragments 2 and 3 resulting in a 1270 bp product. We also generated a larger fragment by ligation of Fragments 4 and 5 forming a 766 bp product. Those two fragments were subjected to another overlap extension PCR reaction to generate a fusion sequence that contained fragments including two to five. Next, the fragments were assembled using molar amounts recommended by the manufacturer with a KAN^R^ -containing vector pC1-CMVp-mScarlet (Addgene # 85042) that was pre-digested with NheI-XhoI to remove the mScarlet ORF. The assembly was transformed to NEB 5-alpha Competent *E. coli* (NEB #C2987). Single KAN^R^-positive transformants were selected and plasmid DNA was purified using QIAprep Spin Miniprep Kit (27104, Qiagen), and the correctly assembled pC1-CMVp-eGFP-ninein^ISO2^: KAN^R^ was confirmed by diagnostic digestion that was followed by Sanger Sequencing at the center for Applied Genomics in The Hospital for Sick Children. Maxi preps were generated with EndoFree Plasmid Kits (12362, Qiagen).

### RT-qPCR analysis

Real-time quantitative RT-PCR (RT-qPCR) amplification was carried out using the primer sets listed in Table 1. In brief, a 20 μl (Fig. 2*B*) or 10 μl (Fig. 2*C*) RT-qPCR reaction mixture containing 10 ng of cDNA was prepared using PowerUp SYBR Green Master Mix (A25741, Thermofisher Scientific) and run on a QuantStudio 3 Real-Time PCR System (A28136, Thermofisher Scientific). Quantification of the expression of ninein^TOT^, ninein^CAN^, and ninein^iso2^ was performed by the amplifying primers qF1/qR1, qF2/qR2, and qF3/qR3, respectively (listed in Table 1).

Following amplification, a melting curve analysis was performed to ensure the specificity of a single amplicon. Threshold cycle (Ct) values for gene expression analysis were obtained using the Relative Quantification Analysis Module, Version 4.3 (Thermofisher Scientific), and expression levels were calculated using the comparative Ct method (2-ΔΔCt). To quantify the amount of ninein^CAN^ and ninein^ISO2^ in the untreated RAW cDNA pool, the geometric mean of Ninein^TOT^ was used to normalize the expression levels of ninein^CAN^ and ninein^ISO2^. Each experiment was conducted with four independent biological replicates. To quantify the amount of ninein transcripts from isoform-depleted and control cDNA pool, the geometric mean of the Beta-glucuronidase (GUSB, RealTime Primers) transcript was used as an internal control to normalize the expression levels of ninein^CAN^ and ninein^ISO2^.

### Immunofluorescence and imaging

For indirect immunofluorescence, cells were either fixed using ice-cold methanol at −20 °C for 10 min or 4% paraformaldehyde for 20 min followed by permeabilization with 0.1% TritonX-100 in PBS containing 100 mM glycine for 20 min. Blocking and antibody dilutions were in PBS containing 3% bovine serum albumin (BSA) (BioShop Canada) and 1% fetal bovine serum (FBS). Epifluorescence images were captured using a Zeiss AxioObserver Z1 inverted microscope equipped with a 63 × 1.4 NA oil immersion lens (Carl Zeiss Canada) and an Axiocam 506 mono-camera, with image acquisition configured *via* Zeiss Zen 3.1 software. Confocal images were obtained using two systems: a Quorum WaveFX-X1 spinning disk confocal microscope (Quorum Technologies Inc) with MetaMorph image acquisition software (Molecular Devices LLC, Sunnyvale, CA) and a 63× oil immersion objective equipped with a cooled electron-multiplying charge-coupled device (EM-CCD) camera (Hamamatsu); and a Leica Stellaris five laser scanning confocal microscope (Leica Microsystems), mounted on an automated Leica DMi8 inverted microscope platform with a white light laser and HyD S detectors, configured with Leica Application Suite X (LAS X) software. Both systems were housed at the Center of Neurobiology of Stress, University of Toronto. For live cell imaging, cells were maintained at 37 °C and 5% CO2.

#### Localization of GFP-fusion constructs

To examine the localization of GFP-fusion constructs, 0.2 to 0.5 × 10^6^ RAW cells were seeded onto coverslips in 24- or 6-well plates and transfected the following day with either GFP-ninein^CAN^ or GFP-ninein^ISO2^ plasmid (1.5 or 2 μg DNA), using FuGENE HD Transfection Reagent (E2311, Promega). Cells were analyzed within 24 h of transfection.

#### MT regrowth assays

These assays were performed by depolymerizing the MT network by treating RAW cells with medium containing DMSO or 10 μM nocodazole for 30 min in siRNA-containing medium at 37 °C and 5% CO_2_. Cells were washed extensively with PBS to remove nocodazole, then incubated with a pre-warmed culture medium to induce MT nucleation at 37 °C and 5% CO_2_. Cells were fixed in methanol at −20 °C for 10 min after one or 3 min of MT regrowth. Cells were immunostained with anti-ninein, anti-Tyr-α-tubulin and anti-γ-tubulin.

#### IgG-opsonization of polystyrene beads and sheep red blood cells (sRBCs)

IgG-opsonization was performed by incubating a pre-washed 100 to 200 μl of 10% suspension sRBCs with 2 μg/ml of anti-sheep IgG in PBS. IgG opsonization using 6.9 μm beads was performed by washing the beads with PBS and opsonizing with 2 mg/ml human IgG at 1:1 ratio in PBS. Both targets (sRBCs and beads) were incubated for 1 h on a rotator at room temperature.

#### Internalization assay

IgG-coated 6.9 μm latex beads were incubated with siRNAs-pretreated RAW cells for 8 min at 37 °C and 5% CO_2_. Cells were then washed twice with prewarmed media to remove unbound beads before cells were further incubated for 12 min in a siRNA-containing medium. Cells were washed with PBS to remove the medium and fixed with 4% PFA in PBS for 20 min. To label bound but not internalized beads, cells were incubated with Cy5 conjugated secondary antibody that was diluted in PBS for 10 min. Cells were then washed with PBS before cell permeabilization with 0.1% TritonX-100 in PBS containing 100 mM glycine for another 20 min. The internalization index was tabulated as the percent of internalized beads divided by the sum of bound and internalized beads.

#### Live cell imaging

For live imaging 0.2 × 10^6^ LifeAct-RFP cells were plated overnight into 35 mm glass-bottom single well dishes (P35G-1.5-14-C, MatTek). The following day, cells were treated with siRNA (as described in the Cell culture and siRNA transfection section) and mounted on a Quorum WaveFX-X1 spinning disk confocal microscope or Zeiss AxioObserver Z1 inverted epifluorescence microscope. Cells were imaged with a 63 × 1.4 NA oil immersion lens and kept at 37 °C and 5% CO_2_ throughout imaging. IgG-sRBCs were added while the RAW cells were in the microscope stage. Time-lapse images were acquired at 16 or 21 s intervals. F-actin dynamics during particle binding and uptake were assessed as described (26). Initial F-actin recruitment was measured from the time when IgG-sRBCs formed firm, non-oscillating contact with the cell surface to the first unambiguous sign of F-actin local presence on the cell surface in close vicinity to the attached particle. To avoid including passive events where IgG-sRBCs non-specifically attach to the surface of RAW cells, F-actin dynamics were only measured for particles that were eventually successfully internalized. The initiation of F-actin recruitment usually coincided with local membrane protrusions or pseudopods around the target particle. Phagocytic events at the periphery of macrophages (*versus* the dorsal surface) were chosen to examine the F-actin cup and pseudopod elaboration.

For assessment of cell proliferation, 0.2 × 10^6^ RAW cells were plated into a 12-well plate in complete DMEM containing 10% heat-inactivated FBS at 37 °C in 5% CO_2_. Cells were treated with ninein^TOT^ or specific siRNA for ∼24 h before a second dose of siRNA treatment was applied. The 12-well plate was then mounted into Etaluma (LS720) automated live cell inverted epifluorescence microscope (Etaluma's LS Microscopes) enclosed within a 37 °C 5% CO_2_ incubator and cells were imaged for 22 h with 20 min interval. Bright-field image sequences were recorded from four ROIs per well and three wells per condition using a 20× dry lens using high sensitivity monochrome CMOS sensor and configured with Lumaview 720/600-Series software (Etaluma's LS Microscopes). To measure the Golgi area, a uniform-sized circle tool in ImageJ was used to encompass GM130 in siRNA-treated cells. The percentage of the number of pixels above the background intensity was determined and divided by the total number of pixels in the selection ROI.

### Immunoblotting

For immunoblotting, siRNA-treated cells were lysed on ice with ice-cold RIPA lysis buffer (10 mM Tris-HCl, 150 mM NaCl, 1% Triton X-100, 0.1% SDS, 1 mM EDTA, pH 7.4) supplemented with protease inhibitor cocktail (P8340, MilliporeSigma) and Laemmli sample buffer (161–0737, BioRad Laboratories Inc). Cell lysates were boiled for 10 min and then separated on 4 to 20% Mini-PROTEAN TGX (4561096, BioRad Laboratories) and transferred to nitrocellulose membranes for 60 to 90 min. Nitrocellulose membranes were then blocked for 1 h in TBST with 5% BSA or for 15 min with EveryBlot Blocking buffer (12010020, BioRad Laboratories) and probed with rabbit anti-ninein (1:300), mouse anti-GAPDH (1:1000), or anti-β actin (1:1000) overnight at 4 °C. Goat horseradish peroxidase (HRP)-conjugated anti-mouse (115-035-146, Jackson ImmunoResearch Laboratories) or goat HRP-conjugated anti-rabbit (711-035-152, Jackson ImmunoResearch Laboratories) were used at 1:10,000 dilutions. Chemiluminescence signals were acquired and imaged using a ChemiDoc Imaging System (BioRad Laboratories). Immunoblots were exposed for durations without saturating the camera's pixels.

### Statistical methods

Data normality was assessed using the Anderson-Darling or D'Agostino-Pearson omnibus tests. For data normally distributed, statistical significance was determined using two-tailed unpaired Student's *t* test or ordinary one-way ANOVA followed by Tukey's or Šídák's multiple comparisons test. For non-normal distributed data, statistical significance was determined using a non-parametric two-tailed Mann U Whitney or Kruskal-Wallis test. Statistical analyses were conducted using Graphpad Prism V9 or above (GraphPad Software, Inc), with *p* < 0.05 considered statistically significant.

## Data availability

All data generated or analyzed during this study are available to share and detailed protocol can be granted through request to the author.

## Supporting information

This article contains supporting information.

## Ethical statement

No animals or human participants.

## Conflict of interest

The authors declare that they have no conflicts of interest with the contents of this article.

## Supporting information

Harrison SUPPLEMENTAL
