# Supplementary material for: Ninein isoform contributions to intracellular processes and macrophage immune function
Source: J Biol Chem. 2025 Mar 18;301(5):108419. doi: 10.1016/j.jbc.2025.108419 (PMC12135376; doi:10.1016/j.jbc.2025.108419)
Supplement: Harrison SUPPLEMENTAL [file mmc1.docx]

**SUPPLEMENTAL FIGURE LEGEND**

**Figure S1. Ninein^CAN^ is expressed in mouse macrophages and shows similarity to human orthologue. (A)** Multiple protein sequence alignments of C-terminus sequences of ninein isoforms in mouse and human. Dashed box highlights the 9 aa motifs in all human isoform 1, 2 and 5 (1951-1959 aa), isoform 6 (1238-1246 aa), and mouse ninein^ISO2^ (1941-1949 aa), ninein^ISO4^ (1941-1949 aa), and ninein^Neuro^ (1234-1242 aa). Note the conserved sequences flanking the 9aa motif. **(B)** Phylogenetic tree of C-terminus sequences of ninein isoforms in mouse and human, generated using maximum likelihood analysis. **(C)** Diagram illustrating the organization of ninein isoforms protein sequences. The binding site of the amplification forward (F) and reverse (R) primers at the corresponding cDNA are indicated as green bars and sequencing primers are shown as magenta bars. **(D)** Multiple DNA sequence alignments showing the Sanger sequencing read of cDNA amplification using F1/R1primers alongside ninein isoforms sequences. Sequencing of F1/R1 reaction was identical to sequences corresponding to ninein^CAN^, ninein^ISO2^, ninein^ISO3^, ninein^ISO4^, but not ninein^Neuro^. Exclusion of ninein^Neuro^ is confirmed by the lack of the sequence corresponding to a central 707 aa encoding exon. **(E and F)** Multiple sequence alignments confirming the presence of ninein^CAN^ in RAW cells. A continuous sequence read of the amplification product from F2/R2 reaction alongside mouse ninein isoforms. Yellow highlight the sequences not matched to the sequencing trace, excluding isoforms containing sequences corresponding to the 9 aa motif; ninein^Neuro^, ninein^ISO2^ and ninein^ISO4^ (E), and exclude isoforms containing the variable C-teminus; ninein^Neuro^, ninein^ISO2^, and ninein^ISO3^ (F).

**Figure S2. Ninein^ISO2^ is expressed in mouse macrophages.**

**(A)** Schematic showing ninein isoforms that contain the 9 aa motif VRLDEKLME at the C-terminus. Amplification (F2/R3) and sequencing primers are shown as green and magenta bars, respectively. Identical protein sequences are depicted in the same color and variable 3'UTR sequences are depicted as dashed vs solid line. **(B-C)** Multiple DNA sequence alignment of a continuous sequence read from a Sanger sequencing reaction. The input sequences were aligned with ninein isoforms containing primer binding sites, including two predicted isoforms XP_006515595 and XM_006515529.3. Sequencing of F2/R3 cDNA amplification product excluded ninein^Neuro^ in (B) and excluded ninein^ISO4^, the two predicted isoforms, and confirmed the presence of sequences specific to ninein^ISO2^ in (C).

**Figure S3. Ninein^ISO2^ shows similar subcellular localization to ninein^CAN^.**

**(A)** Schematic representation of N-terminally GFP-tagged ninein^CAN^ and GFP-tagged ninein^ISO2^ constructs.. **(B)** Confocal images of RAW cells transiently transfected with GFP-ninein^CAN^ and GFP-ninein^ISO2^, fixed, and immunostained with anti-GFP and anti-ninein antibodies. White arrowheads indicate non-transfected cells, while yellow arrowheads mark transfected cells. **(C)** RAW cells transiently transfected with GFP-ninein^CAN^ and GFP-ninein^ISO2^, fixed and immunostained with anti-GFP and anti-γ-tubulin antibodies. **(D)** Quantification of γ-tubulin enrichment at the centrosome in GFP-ninein^CAN^- and GFP-ninein^ISO2^-transfected cells, compared to non-transfected control cells (n ≥ 11). p-values were calculated using one-way ANOVA followed by Tukey’s multiple comparisons test. Error bars represent SEM (D). **(E)** Confocal images of RAW cells transfected with GFP-ninein^CAN^ and GFP-ninein^ISO2^, fixed and immunostained with anti-GFP and anti-α-tubulin antibodies. White, yellow, and magenta arrowheads indicate medium expressors, low expressors, and non-transfected cells, respectively. The number of samples and statistical significance of MT level changes in transfected cells compared to controls are indicated. p-values were calculated using one-way ANOVA followed by Tukey’s multiple comparisons test. Scale bars = 10 µm.
